# Supplementary material for: Skeletal stem and progenitor cells maintain cranial suture patency and prevent craniosynostosis
Source: Nat Commun. 2021 Jul 30;12:4640. doi: 10.1038/s41467-021-24801-6 (PMC8324898; doi:10.1038/s41467-021-24801-6)

Supplementary Figure 1

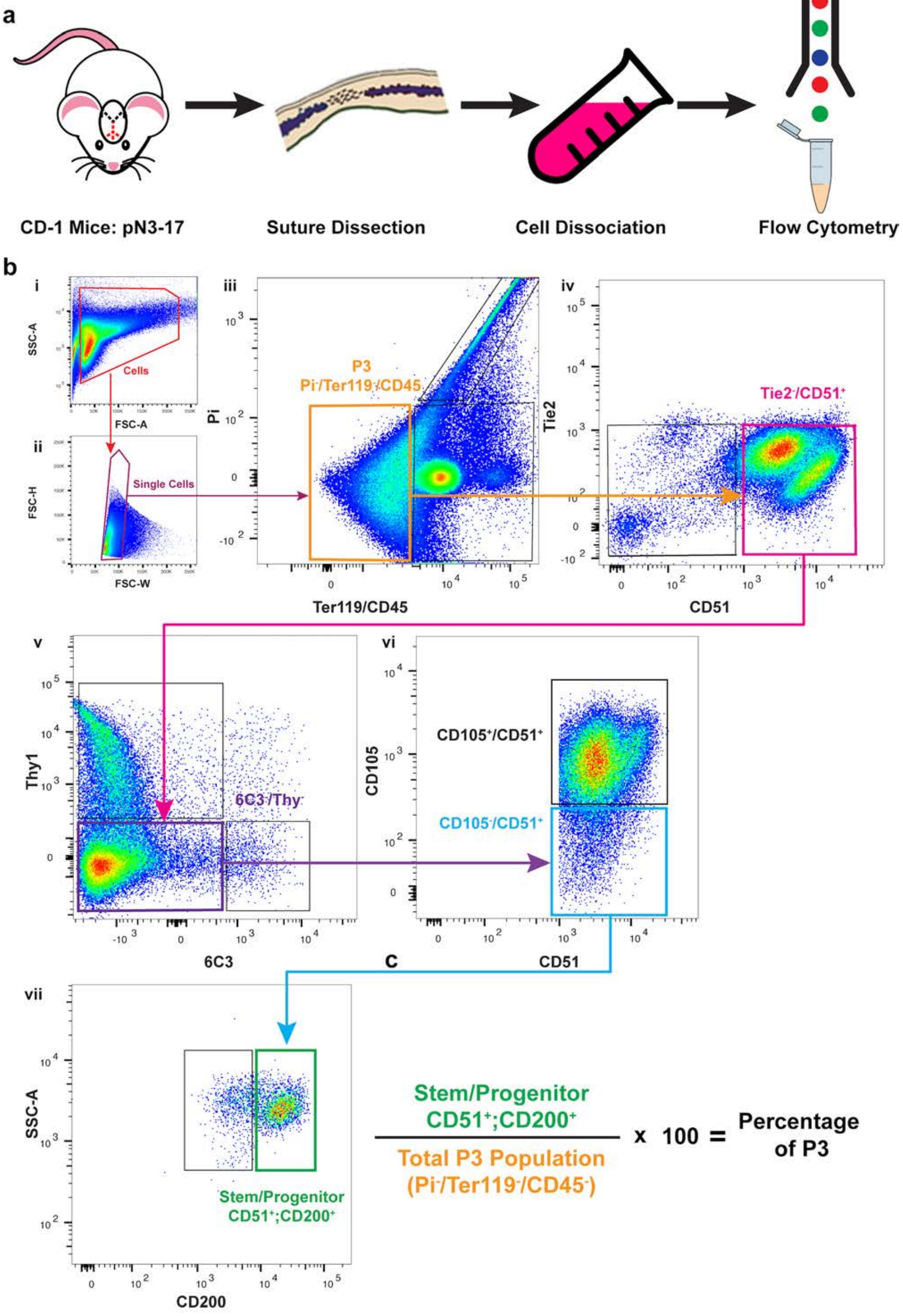

Supplementary Figure 1. Gating Strategy for Isolation of CD51$^+$;CD200$^+$ Cells from Cranial Sutures. a, Schematic representation of cranial suture harvest and fractionation by flow cytometry. pN3-17 CD-1 mice cranial sutures were dissected including the entire suture complex. Whole tissue was dissociated by mechanical and enzymatic digestions. Dissociated cells were stained with fluorochrome-conjugated antibodies Ter119, CD51, CD105, Thy1.1, Thy1.2, Tie2, CD45, 6C3, CD200 and fractionated by flow cytometry. **b,** Representative FACS plots from pN3 CD-1 mice cranial sutures. **i** and **ii.** Total dissociated cells were fractionated by side scatter (SSCs) and forward scatter (FSC) to discriminate single cells vs debris and doublets. **iii.** Single cells were then fractionated by Pi (viability marker), Ter119 and CD45 into the P3 population (Pi$^-$/Ter119$^-$/CD45$^-$). **iv.** The P3 population was then further fractionated by Tie2 and CD51. **v.** Tie2$^-$/CD51$^+$ cell population was gated for Thy1 and 6C3. **vi.** The double negative, Thy1$^-$/6C3$^-$ population was next gated against CD105. **vii.** CD105$^-$/CD51$^+$ population was gated against CD200 for final isolation of skeletal stem/progenitor cells (CD51$^+$;CD200$^+$): CD45$^-$, Ter119$^-$, Tie2$^-$, Thy1.1$^-$, Thy1.2$^-$, 6C3$^-$, CD105$^-$, **CD51$^+$**, **CD200$^+$**. **c,** Calculation of percentage of P3. Total number of CD51$^+$;CD200$^+$ skeletal stem/progenitor cells was normalized to the P3 population (Pi$^-$/Ter119$^-$/CD45$^-$) for comparison among the three cranial sutures and various treatment groups. This gating strategy was used for isolation of CD51$^+$;CD200$^+$ skeletal stem/progenitor cells in Figure 1b&d, 3b&g, 4c&d, 5b, and Supplementary Figure 7c.

Supplementary Figure 2

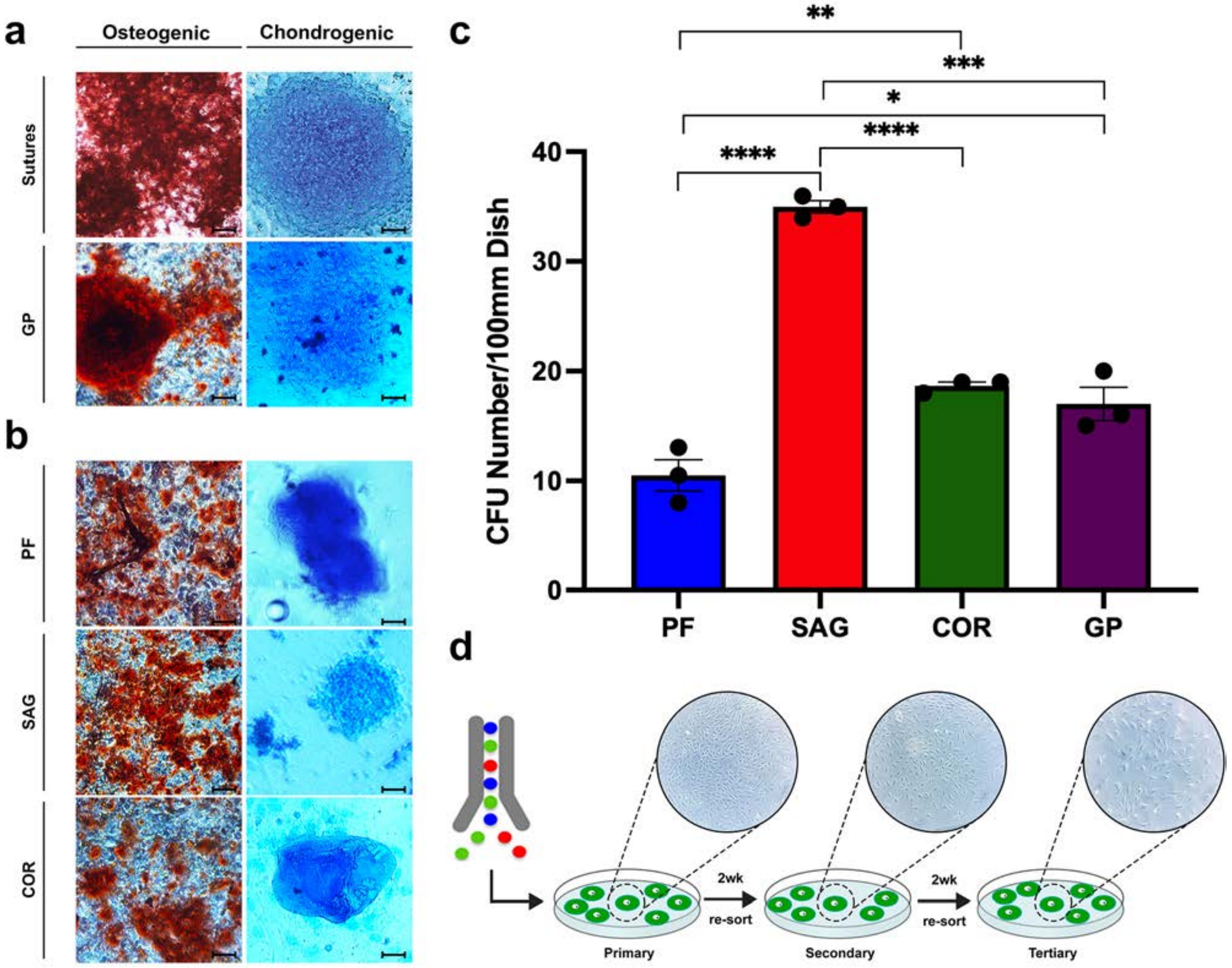

Supplementary Figure 2. Lineage Differentiation of Sutures-Derived CD51$^+$;CD200$^+$ Cells. a, Representative osteogenic differentiation assay performed on a pool of CD51$^+$;CD200$^+$ cells isolated from PF, SAG and COR sutures (left, top panel) and from the GP (left, bottom panel) at day pN3. CD51$^+$;CD200$^+$ cells were seeded at a density of 3x10^3 cells per well. (Pooled cells from the sutures comprised of 1x10^3 CD51$^+$;CD200$^+$ cells per suture.) Alizarin red staining carried out after 21 days detected a robust mineralization of the extracellular matrix. Representative chondrogenic differentiation assay performed on CD51$^+$;CD200$^+$ cells derived from the entire skull (right, top panel) and GP (right, bottom panel). Alcian blue staining at day 40 revealed strong staining for proteoglycans in micro-masses formed by CD51$^+$;CD200$^+$ cells isolated from the skull and GP, seeded at 1x10^5 cells per well. Scale bar, 150 µm. n=60animals/group, experiments were repeated 3 independent times. **b,** Representative osteogenic (left panel) and chondrogenic (right panel) differentiation assays were performed on CD51$^+$;CD200$^+$ cells isolated from the PF, SAG and COR sutures independently at a seeding density of 1x10^3 cells/well. Alizarin red staining carried out after 21 days detected mineralization of the extracellular matrix and light Alcian blue staining for proteoglycans. Scale bar, 150 µm. n=60animals/group, experiments were repeated 3 independent times. **c**, Colony forming units assay (CFU) performed on CD51$^+$;CD200$^+$ cells freshly isolated from the PF, SAG and COR sutures compared to the GP. Values represent mean ± SEM; PF vs SAG P= <0.0001, SAG vs COR P = <0.0001, PF vs COR P =0.0053, PF vs GP P =0.0365, SAG vs GP P =0.0004; * $P \leq 0.05$, **$P \leq 0.01$, ***$P \leq 0.001$, ****$P \leq 0.0001$ unpaired, two-tailed student t-test were performed. n=60 animals/group, experiments were repeated 3 independent times. **d**, FACS isolation of CD51$^+$;CD200$^+$ cells for evaluation of serial colony-forming potential. CD51$^+$;CD200$^+$ cells from clones were isolated by FACS and passaged three times. Primary, (left panel); secondary, (middle panel); tertiary, (right panel). CD51$^+$;CD200$^+$ cells were able to form secondary colonies with an efficiency of 1.35% and tertiary colonies with an efficiency of .99%. 'Efficiency' is calculated as a percentage of cells seeded over cells recovered. Source data are provided as a Source Data file.

Supplementary Figure 3

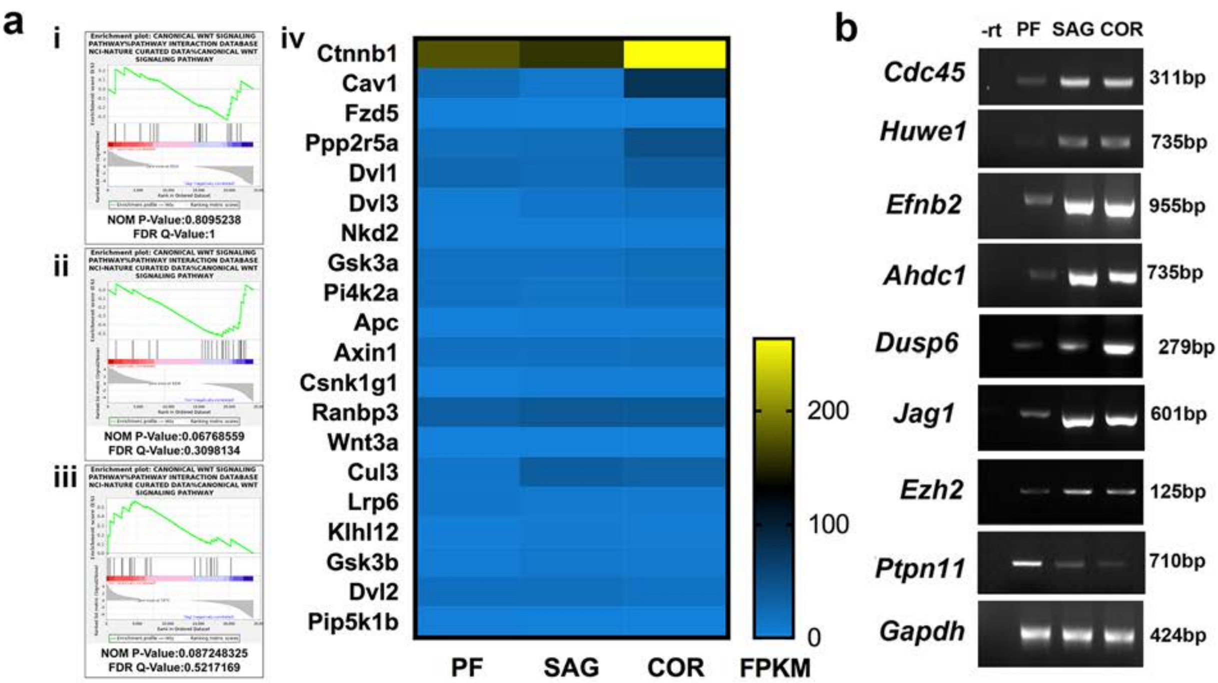

c

CDC45	Fenwick, A.L., et al., *Mutations in CDC45, Encoding an Essential Component of the Pre-initiation Complex, Cause Meier-Gorlin Syndrome and Craniosynostosis.* Am J Hum Genet, 2016
HUWE<br>EFNB1<br>AHDC1	Miller, K.A., et al., *Diagnostic value of exome and whole genome sequencing in craniosynostosis.* J Med Genet, 2017.
DUSP6	LI, C. et al. *Dusp6 (Mkp3) is a negative feedback regulator of FGF-stimulated ERK signaling during mouse development,* Development, 2007.
JAG	Kamath, B.M., et al., *Craniosynostosis in Alagille syndrome.* Am J Med Genet, 2002.
EZH2	Dudakovic, A., et al., *Epigenetic Control of Skeletal Development by the Histone Methyltransferase Ezh2.* J Biol Chem, 2015.
PTPN11	Pannone, L., et al., *Structural, Functional, and Clinical Characterization of a Novel PTPN11 Mutation Cluster Underlying Noonan Syndrome.* Hum Mutat, 2017.

Supplementary Figure 3. cWnt GSEA and Validation of Bulk RNA-seq Analysis of Genes Associated with Craniosynostosis. a, Gene Set Enrichment Analysis (GSEA) was performed on bulk-RNA sequencing of $CD51^{+}$;$CD200^{+}$ cells isolated from pN3 PF, SAG, and COR sutures. GSEA results for the cWnt pathway shows no significant enrichment at pN3 across the three sutures. **i,** PF vs. SAG for SAG; **ii**, PF vs COR for COR; **iii,** COR vs SAG for COR. Bar-codes indicate the positions of each gene within the set; red and blue colors represent positive and negative Pearson correlations, respectively. **iv**, Heat map of FPKM counts from cuffdiff analysis of bulk-RNA sequencing of $CD51^{+}$;$CD200^{+}$ cells isolated from pN3 PF, SAG, and COR sutures, demonstrates no significant difference in counts among the gene set identified by GSEA of the Canonical Wnt Pathway. Yellow: high counts; blue: low counts. **b**, RT-PCR analysis confirmed the unique expression of selected genes in PF-$CD51^{+}$;$CD200^{+}$ cells relatively to cells isolated either from the SAG or COR sutures. $CD51^{+}$;$CD200^{+}$ cells were isolated by FACS from the PF, SAG, and COR sutures. n=60animals/group, experiments were repeated 1 time. **c,** Table listing genes associated to human craniosynostosis and/or cranial suture fusion in mouse models.

Supplementary Figure 4

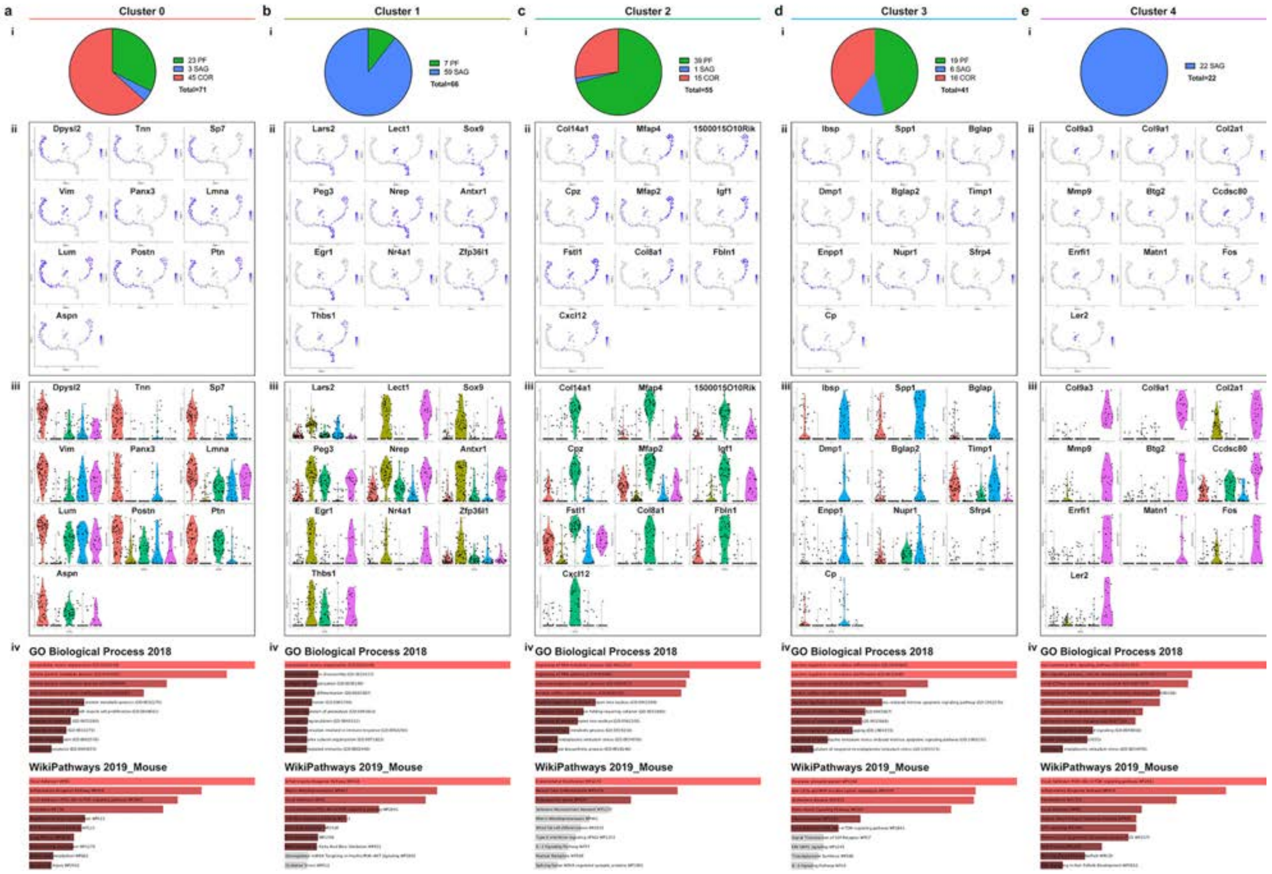

Supplementary Figure 4. Additional scRNA-seq Analysis of the PF, SAG, and COR Sutures. a-e, scRNA-seq analysis of clusters 0 - 4 identified in **Figure 2d-e**. **i,** Pie chart showing the composition of each SSC cluster isolated from the 3 cranial sutures. **ii,** Feature plots of the top 10 gene features defining each cluster. **iii,** Violin Plots of the top 10 gene features defining each cluster. **iv,** Gene Ontology (top panel) and pathway analysis (bottom panel) analysis using the top 50 gene features defining each cluster with the EnrichR gene enrichment analysis tool (https://amp.pharm.mssm.edu/Enrichr/).

Supplementary Figure 5

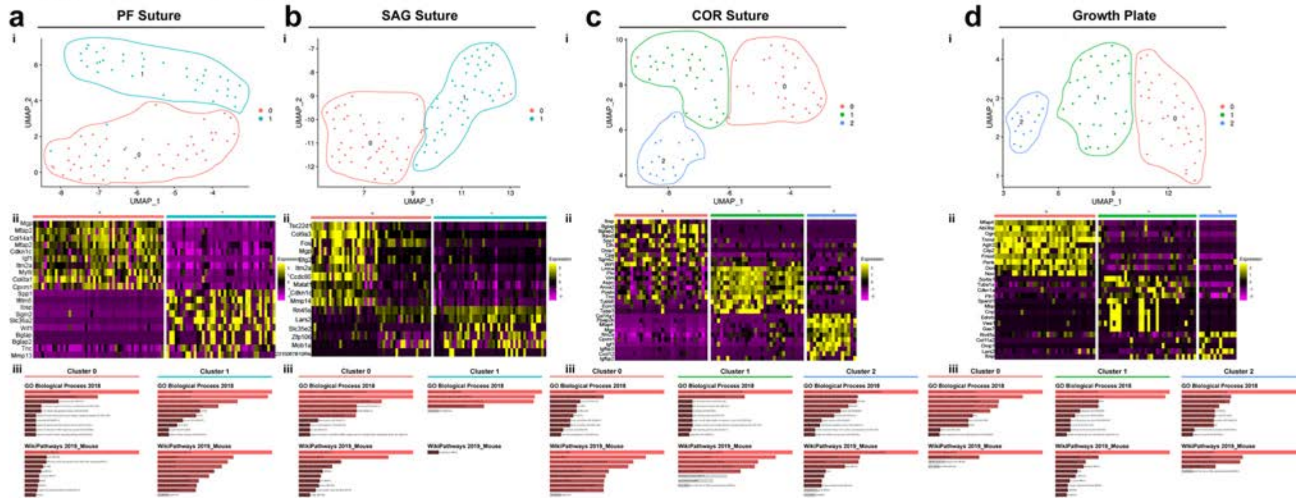

Supplementary Figure 5. Subset Analysis of CD51$^+$;CD200$^+$ scRNA-seq Data Derived from the PF, SAG, and COR Sutures and Growth Plate (GP). a, UMAP embedding showing the distribution of transcriptional programs for single CD51$^+$;CD200$^+$ cells isolated from the PF suture **i,** Two transcriptionally-defined clusters were identified among CD51$^+$;CD200$^+$ cells isolated from the PF suture. **ii,** Heatmap of the top 10 differentially expressed genes defining each cluster. **iii,** Gene Ontology (top panel) and pathway analysis (bottom panel) analysis of the top 50 gene features defining each cluster pathway using the Enrichr gene list enrichment analysis tool (https://amp.pharm.mssm.edu/Enrichr/). **b**, UMAP embedding showing the distribution of transcriptional programs for single CD51$^+$;CD200$^+$ cells isolated from the SAG suture. **i,** PCA clustering identified 2 clusters among CD51$^+$;CD200$^+$ cells isolated from the SAG suture. **ii,** Heatmap of the top 10 differentially expressed genes defining each cluster. **iii,** Gene Ontology (top panel) and pathway analysis (bottom panel) analysis of the top 50 gene features defining each cluster pathway using the Enrichr gene list enrichment analysis tool (https://amp.pharm.mssm.edu/Enrichr/). **c**, UMAP embedding showing the distribution of transcriptional programs for single CD51$^+$;CD200$^+$ cells isolated from the COR suture **i,** PCA clustering identified 3 clusters among CD51$^+$;CD200$^+$ cells isolated from the COR suture. **ii,** Heatmap of the top 10 differentially expressed genes defining each cluster. **iii,** Gene Ontology (top panel) and pathway analysis (bottom panel) analysis of the top 50 genes features defining each cluster pathway using the Enrichr gene list enrichment analysis tool (https://amp.pharm.mssm.edu/Enrichr/). **d**, UMAP embedding showing the distribution of transcriptional programs for single CD51$^+$;CD200$^+$ cells isolated from the growth plate. **i,** UMAP clustering identified 3 clusters among CD51$^+$;CD200$^+$ cells isolated from the growth plate. **ii,** Heatmap of the top 10 differentially expressed genes defining each cluster. **iii,** Gene Ontology (top panel) and pathway analysis (bottom panel) analysis of the top 50 gene features defining each cluster pathway using

Supplementary Figure 6

a

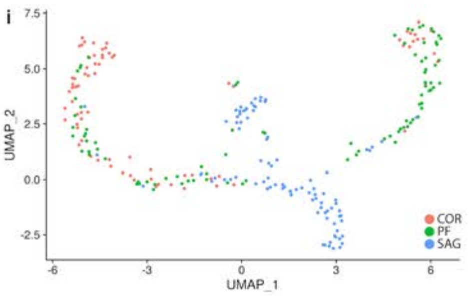

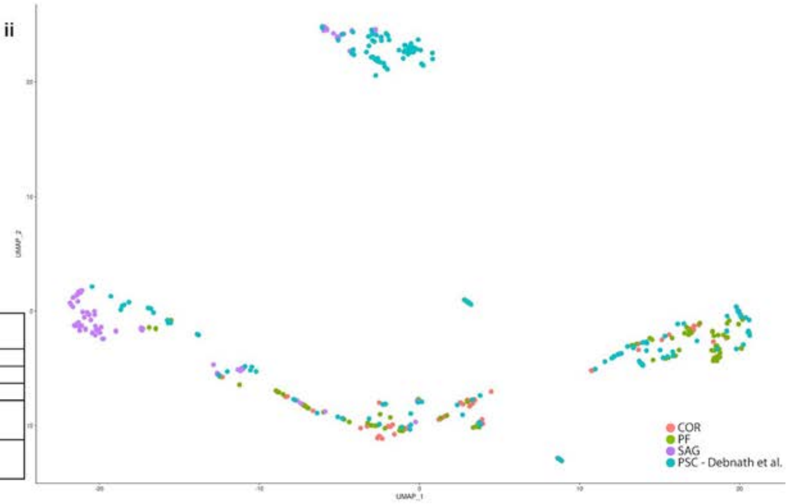

b

Data Set	Tissue Source/ Subpopulation	ROGUE Score
Menon et al. (GSE138882)	PF-CD51$^+$;CD200$^+$ Cells	0.916643
	SAG-CD51$^+$;CD200$^+$ Cells	0.8733708
	COR-CD51$^+$;CD200$^+$ Cells	0.856805
Chan et al., Cell 2015 (GSE64447)	SSC	0.911272
Debnath et al., Nature 2018 (GSE106237)	PSC	0.848873

Supplementary Figure 6. scRNA-seq Analysis of CD51$^+$;CD200$^+$ Cells Derived from the PF, SAG, and COR Sutures in Comparison to Periosteal Skeletal Stem Cells a, Comparison of suture derived CD51$^+$;CD200$^+$ cells and Periosteal Stem Cells (PSC). **i,** UMAP embedding showing the distribution of transcriptional programs for single CD51$^+$;CD200$^+$ cells isolated from the PF, SAG, and COR sutures. Cells are colored by suture of origin. **ii**, UMAP embedding of scRNA-seq data for PF, SAG, and COR Suture-CD51$^+$;CD200$^+$ cells combined with publicly available scRNA-seq data for Periosteal Stem Cells (PSCs) from *Debnath et al.,* using Seurat's anchor-based cross-platform integration technique.[45] Cells are colored by origin. **b,** Entropy-based purity assessment of scRNA-seq samples using the Ratio of Global Unshifted Entropy (ROGUE) toolkit. Purity scores range from 0 (maximal heterogeneity) to 1 (putatively "pure") based on differences between observed and expected entropy.[46]

Supplementary Figure 7

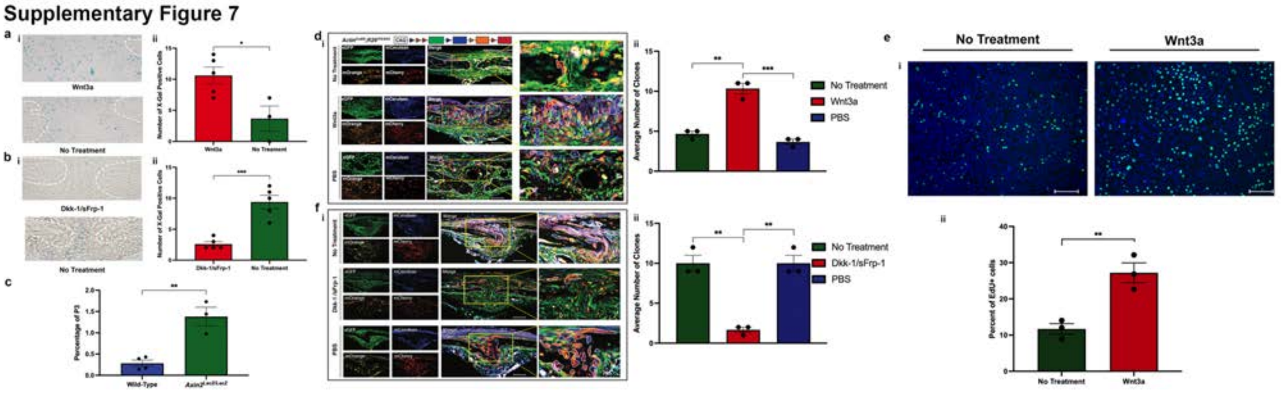

Supplementary Figure 7. **Activation and/or Inhibition of Endogenous cWnt Signaling in the PF and SAG Sutures.** **a**, Validation of activation of cWnt signaling. **i,** X-gal staining performed on PF sutures harvested from *Axin2*$^{LacZ/+}$ mice treated with Wnt3a (150 ng) confirmed activation of Wnt signaling. **ii**, Quantification of positive X-gal stained cells obtained. Values represent mean ± SEM, *P* =0.0254, **P* ≤0.05, unpaired, two-tailed student t-test were performed. n=3 animals/group, experiments were repeated 3 independent times. **b**, Validation of inhibition of cWnt signaling. **i,** X-gal staining of *Axin2*$^{LacZ/+}$ SAG sutures treated with cWnt signaling inhibitors sFrp1 (2µg) and Dkk1 (2µg). **ii**, Quantification of positive X-gal stained cells obtained. Values represent mean ±SEM; *P* =0.0004, ****P*≤0.001, unpaired, two-tailed student t-test were performed. n=3 animals/group, experiments were repeated 3 independent times. **c,** FACS analysis of CD51^{+};CD200^{+} cells isolated from wild-type and *Axin2* $^{LacZ/LacZ}$ PF sutures at pN15. Values represent mean ±SEM; *P* =0.003, ** *P* ≤0.01, unpaired, two-tailed student t-test were performed. n=15 animals/group, experiments were repeated 4 independent times. **d, i,** Confocal micrographs of Wnt3a-treated *ActinCre*^{ERT2}:*Rainbow*$^{+/+}$ PF sutures. Scale bars: 100 µm. Magnification at 20X. n=3 animals/group, experiments were repeated 3 independent times **ii,** Quantification of confocal micrographs of Wnt3a-treated *ActinCre*^{ERT2}:*Rainbow*$^{+/+}$. Values represent mean ± SEM, No Treatment vs Wnt3a *P* =0.0016, PBS vs Wnt3a *P* =0.0009, ***P*≤0.01, ****P*≤0.001, unpaired, two-tailed student t-test were performed. n=3 animals/group, experiments were repeated 3 independent times. **e,** Wnt3a proliferation assay. **i.** Control (left panel), Wnt3a treatment (right panel). Scale bar, 50µm, n=60animals/group, experiments were preformed 3 independent times. **ii,** Quantification of proliferating cells. Values represent mean ± SEM, *P*=0.0075, ***P*≤0.01, unpaired, two-tailed student t-test were performed. n=60 animals, experiments were performed 3 independent times. **f**, **i,** Confocal micrographs of sFrp1 and Dkk1 treated versus untreated PF sutures from *ActinCre*^{ERT2}:*Rainbow*$^{+/+}$ mice. Scale bars: 100 µm. Magnification at 20X. n=3 animals/group, experiments were repeated 3 independent times. **ii,** Quantification of confocal micrographs of Dkk-1/sFRp-1 treated *ActinCre*^{ERT2}:*Rainbow*$^{+/+}$ SAG sutures. Values represent mean ± SEM, No Treatment vs Dkk-1/sFRP-1 *P*=0.0014, Dkk-1/sFRP-1 vs PBS *P*=0.0014, ***P*≤0.01, unpaired, two-tailed student t-test were performed. n=3 animals/group, experiments were repeated 3 independent times. Source data are provided as a Source Data file.

Supplementary Figure 8

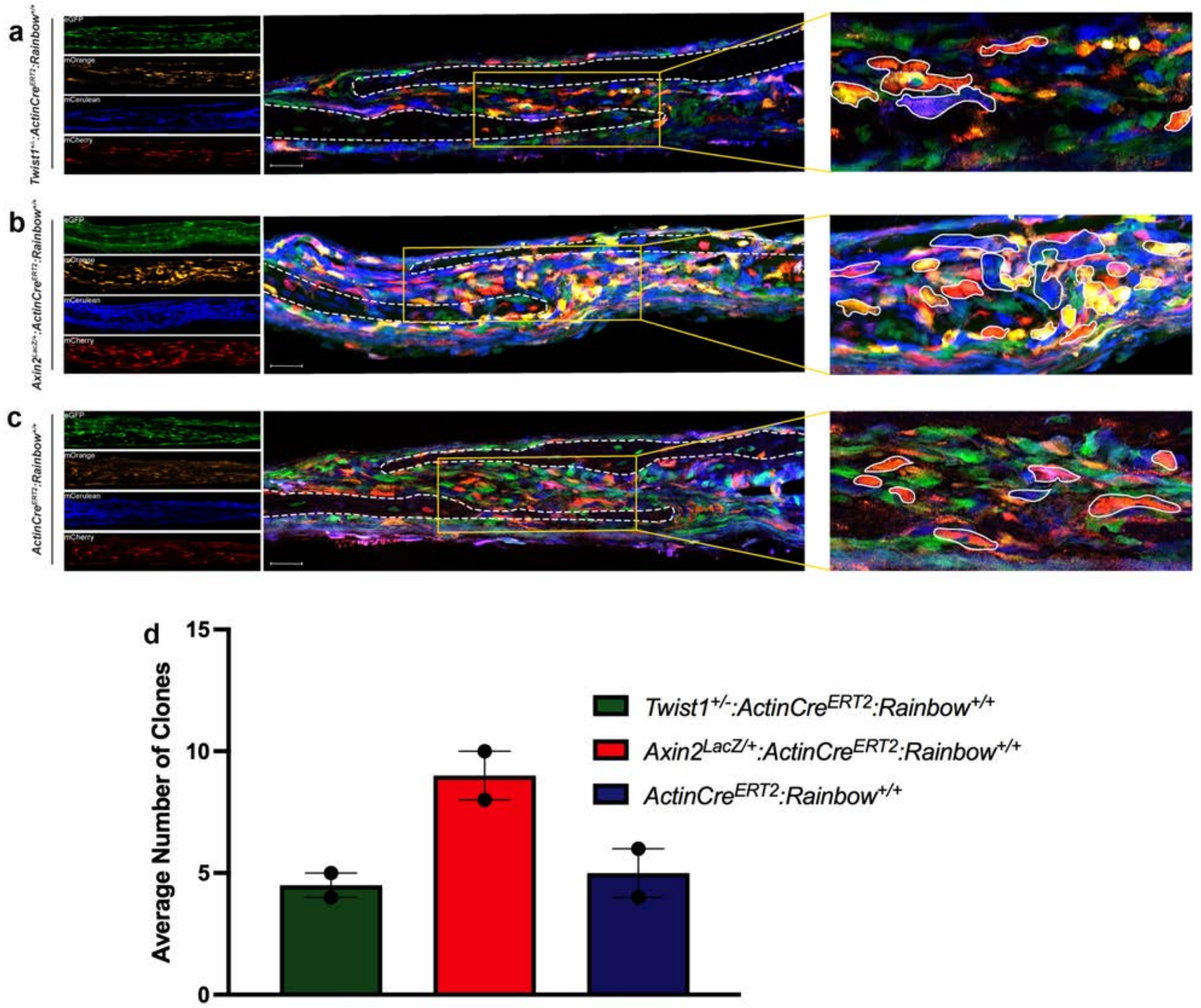

Supplementary Figure 8. Reduced Clonality in *Twist1*$^{+/}$*:ActinCre*^{ERT2}*:Rainbow*$^{+/+}$ COR Sutures Conversely Parallels to Increased clonality in *Axin*$^{LacZ/+}$*:ActinCre*^{ERT2}*:Rainbow*$^{+/+}$. a, Confocal micrographs of pN7 COR suture from *Twist1*$^{+/-}$:*ActinCre*^{ERT2}:*Rainbow*$^{+/+}$ showing decreased clonality in comparison to *Axin*$^{LacZ/+}$:*ActinCre*^{ERT2}:*Rainbow*$^{+/+}$ (**b**) and wild-type *ActinCre*^{ERT2}:*Rainbow*$^{+/+}$ (**c**). **b**, In contrast, a larger number of clones are present in COR suture harvested from *Axin2*$^{LacZ/+}$:*ActinCre*^{ERT2}:*Rainbow*$^{+/+}$ mice as compared to *Twist1*$^{+/-}$:*ActinCre*^{ERT2}:*Rainbow*$^{+/+}$ and wild-type *ActinCre*^{ERT2}:*Rainbow*$^{+/+}$ as well. **c**, COR suture from wild-type *ActinCre*^{ERT2}:*Rainbow*$^{+/+}$ displays an intermediate clonality between that of COR sutures derived from *Twist1*$^{+/-}$:*ActinCre*^{ERT2}:*Rainbow*$^{+/+}$ and *Axin2*$^{LacZ/+}$:*ActinCre*^{ERT2}:*Rainbow*$^{+/+}$ mice. Mice were induced at day pN3 with 50µl (20mg/ml) tamoxifen delivered subcutaneously, whole skulls were harvest on pN7 and sagittal sections of the COR suture were cut and imaged for analysis. Scale bars: 100 µm. Magnification 20x. n=3 animals/group, experiments were performed two independent times. **d**, Quantification of *Twist1*$^{+/-}$:*ActinCre*^{ERT2}:*Rainbow*$^{+/+}$, *Axin2*$^{LacZ/+}$:*ActinCre*^{ERT2}:*Rainbow*$^{+/+}$ and wild-type *ActinCre*^{ERT2}:*Rainbow*$^{+/+}$ COR sutures at pN7 showing decreased clonality in *Twist1*$^{+/-}$:*ActinCre*^{ERT2}:*Rainbow*$^{+/+}$ COR sutures. n=3 animals/group, experiments were performed two independent times. Values represent mean ± SEM. Source data are provided as a Source Data file.

Supplementary Figure 9

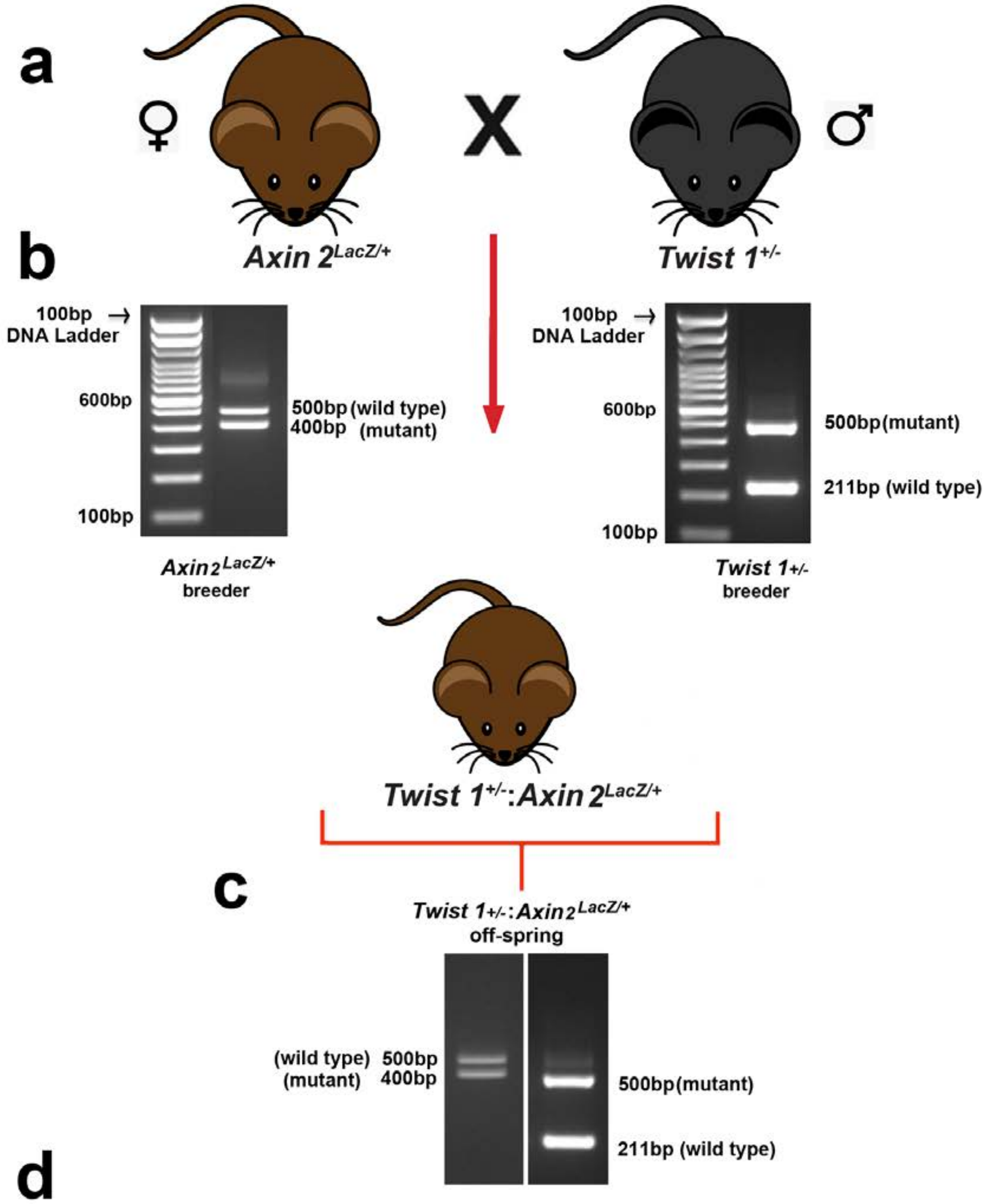

d

Number of Litters (day pN6-6month)	Average Litter Size	Male/Female Distribution	Genotype Distribution	
25	9	Male: 51 %<br>Female: 48 %	Wild Type	12 %
			Axin2$^{LacZ/+}$	13 %
			Twist1$^{+/-}$	42%
			Twist1$^{+/-}$:*Axin2*$^{LacZ/+}$	32%

Supplementary Figure 9. Schematic Representation of Double *Twist1*$^{+/-}$*:Axin2*$^{LacZ/+}$ Transgenic mouse. a, Breeding scheme to generate *Twist1*$^{+/-}$*:Axin2*$^{LacZ/+}$ obtained by crossing a female *Axin2*$^{LacZ/+}$ mouse on a mixed background (CD-1/C57-Black6) with a male *Twist1*$^{+/-}$ on the Black-6 background. **b**, Genotyping of breeders identified by PCR analysis identified the *Axin2*$^{LacZ/+}$ mouse with a ~500bp (wild-type) and ~400bp (mutant) bands and the *Twist1*$^{+/-}$ mouse with a ~500bp (mutant) and 221bp (wild-type) bands. **c**, Genotype of *Twist1*$^{+/-}$*:Axin*$^{LacZ/+}$ off-spring performed through two independent PCR analysis (using either *Axin2* or *Twist1* primers) to confirm the generation of a *Twist1*$^{+/-}$*:Axin*$^{LacZ/+}$ double transgenic mouse. **d**, Summary of number of mice litters screened from day pN6 to 6 months, and their sex and genotype distributions.

Supplementary Figure 10

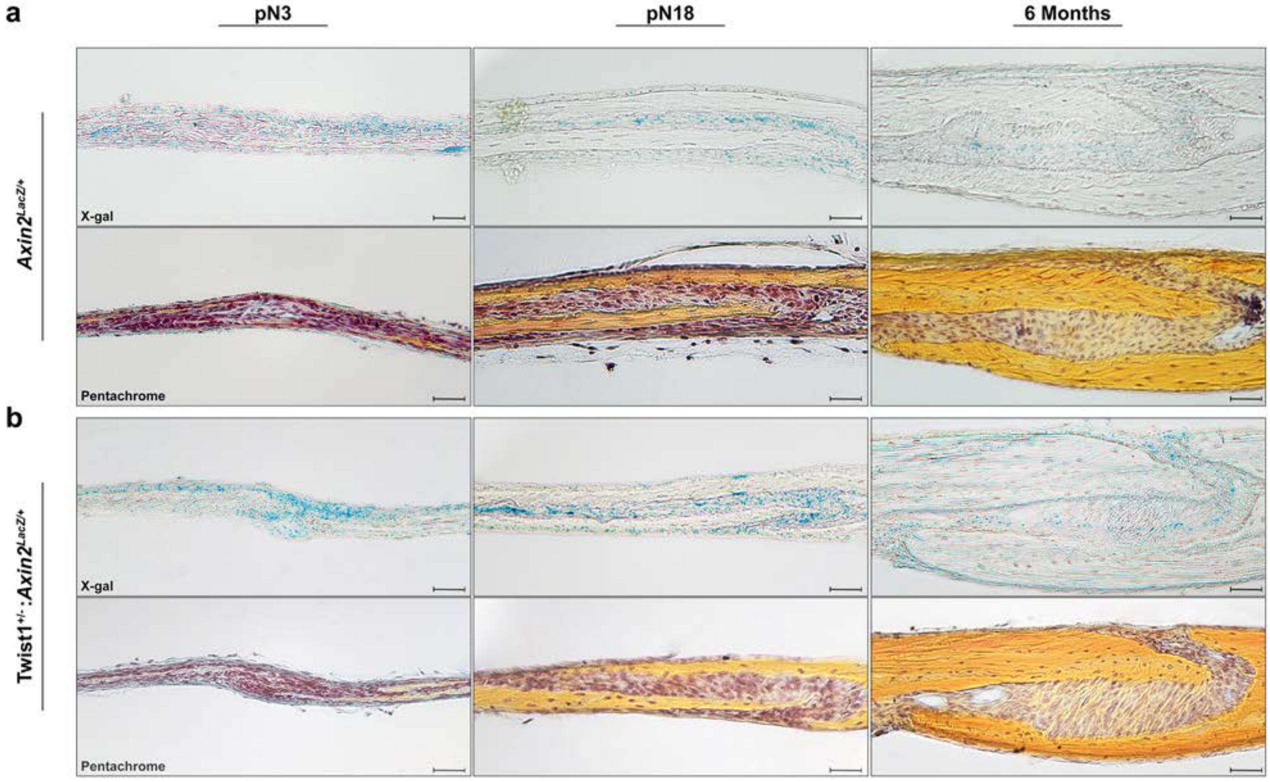

Supplementary Figure 10. Comparative Analysis of Activation of cWnt Signaling in Axin2$^{LacZ/+}$ and *Twist1$^{+/-}$* :*Axin2$^{LacZ/+}$* COR sutures. a, Sagittal sections of COR sutures from *Axin2$^{LacZ/+}$* mice at day pN3, pN18, and 6 months postnatal. (top panel) X-gal staining of the patent COR suture from *Axin2$^{LacZ/+}$* mice. (bottom panel) Movat's pentachrome staining performed on adjacent slide sections as for top panels. n=3animals/group, experiments were repeated 3 independent times. **b,** Sagittal sections of COR sutures from *Twist1$^{+/-}$*:*Axin2$^{LacZ/+}$* mice at day pN3, pN18, and 6 months postnatal. (top panel) X-gal staining of the patent COR suture from *Twist1$^{+/-}$*:*Axin2$^{LacZ/+}$* mice. (bottom panel) Movat's pentachrome staining performed on adjacent slide sections as for top panels. X-gal staining identifies a similar contribution of cWnt-activated *Axin2^{+}* cells within the patent COR suture mesenchyme in both *Axin2$^{LacZ/+}$*and *Twist1$^{+/-}$*: *Axin2$^{LacZ/+}$* double transgenic mice at day pN3, pN18, and 6 months postnatal. The comparable contribution of cWnt active, *Axin2^{+}* cells, highlights the importance of a suture mesenchyme active in cWnt signaling to maintaining patency, thus, preventing the fusion normally observed in a *Twist1$^{+/-}$* mouse COR suture. Magnification 20x, scale bar: 100µm. n=3animals/group, experiments were repeated 3 independent times.

Supplementary Figure 11

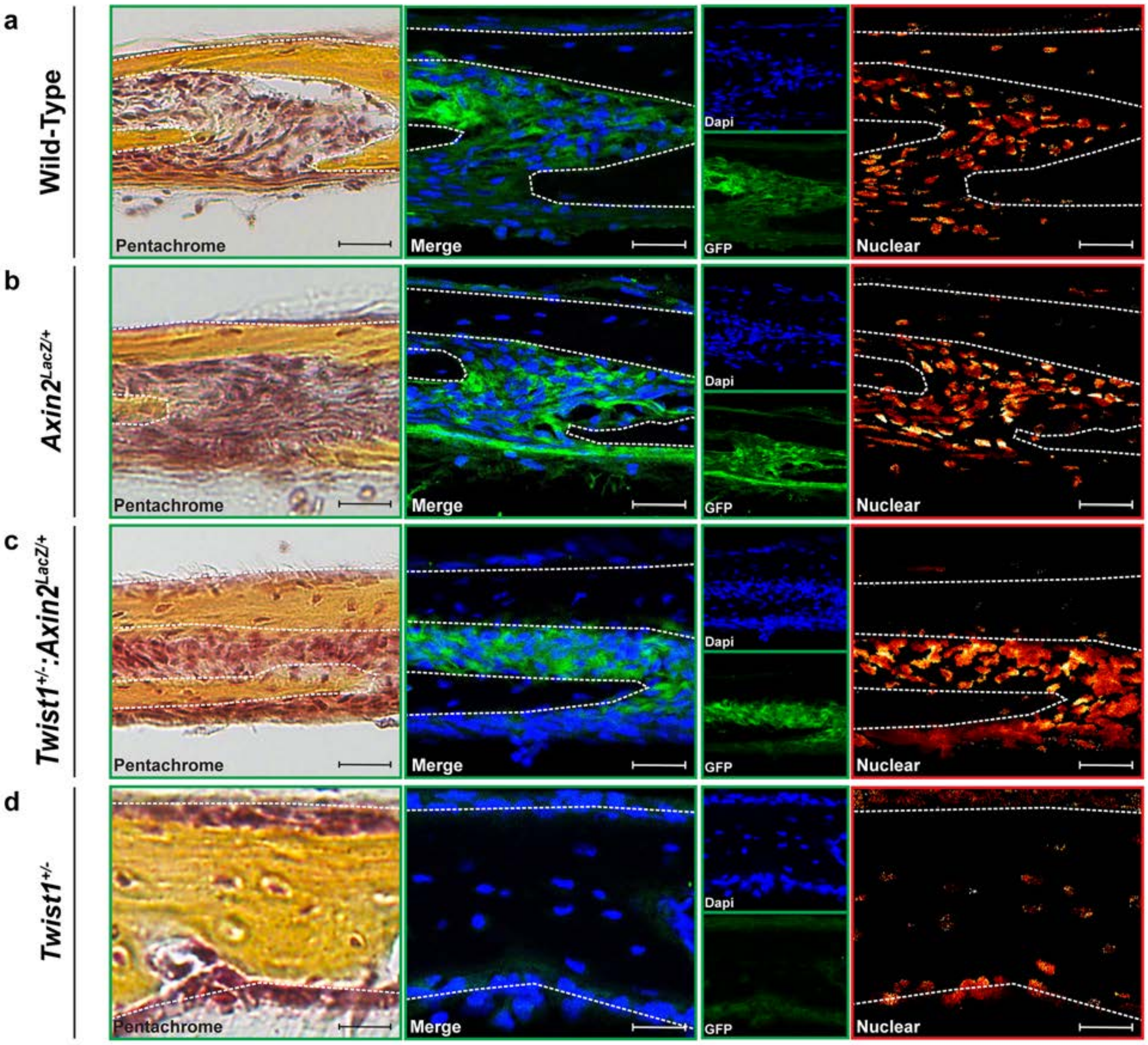

Supplementary Figure 11. Immunofluorescence Staining for Activated β-Catenin. Activation of β-Catenin in **a**, Wild-Type; **b,** *Axin2*$^{LacZ/+}$; **c,** *Twist1*$^{+/-}$:*Axin2*$^{LacZ/+}$; **d,** *Twist1*$^{+/-}$ COR sutures at pN15. (left panel), Movat's pentachrome staining of sagittal sections of COR sutures at pN15. Dotted lines outline the osteogenic bone fronts. (middle panel), confocal micrographs of a sister sections of COR sutures at pN15, stained with a specific activated anti-β-Catenin antibody. GFP signal indicates active β-catenin within the suture complex. Dotted lines outline the osteogenic bone fronts. (right, red box) To assess the degree of nuclear localization of β-Catenin, we implemented the EzColocalization plugin on ImageJ to map the cells within the suture mesenchyme in which β-Catenin has translocated to the nucleus by mapping the colocalization of DAPI (nuclear stain) and GFP (β-catenin). The accompanying heatmap from EzColocalization is shown for each group. High intensity, yellow colors, represent areas of colocalization of DAPI and GFP, indicating nuclear β-Catenin. **a,** immunofluorescence staining for endogenous activated β-Catenin in wild-type COR suture. **b**, immunofluorescence staining shows increased active β-Catenin in the patent *Axin2*$^{LacZ/+}$ COR suture in the suture mesenchyme and underlying dura-mater as compared to wild-type (**a**) and *Twist1*$^{+/-}$:*Axin2*$^{LacZ/+}$ COR sutures (**c**), which both display similar activation. **d**, *Twist1*$^{+/-}$, fused COR suture showed little to no active β-Catenin. These data support the critical role of active cWnt signaling within the suture mesenchyme in maintaining suture patency. Magnification 20x, scale bar: 100µm. n=3 animals/group, experiments were performed three independent times.

Supplementary Figure 12

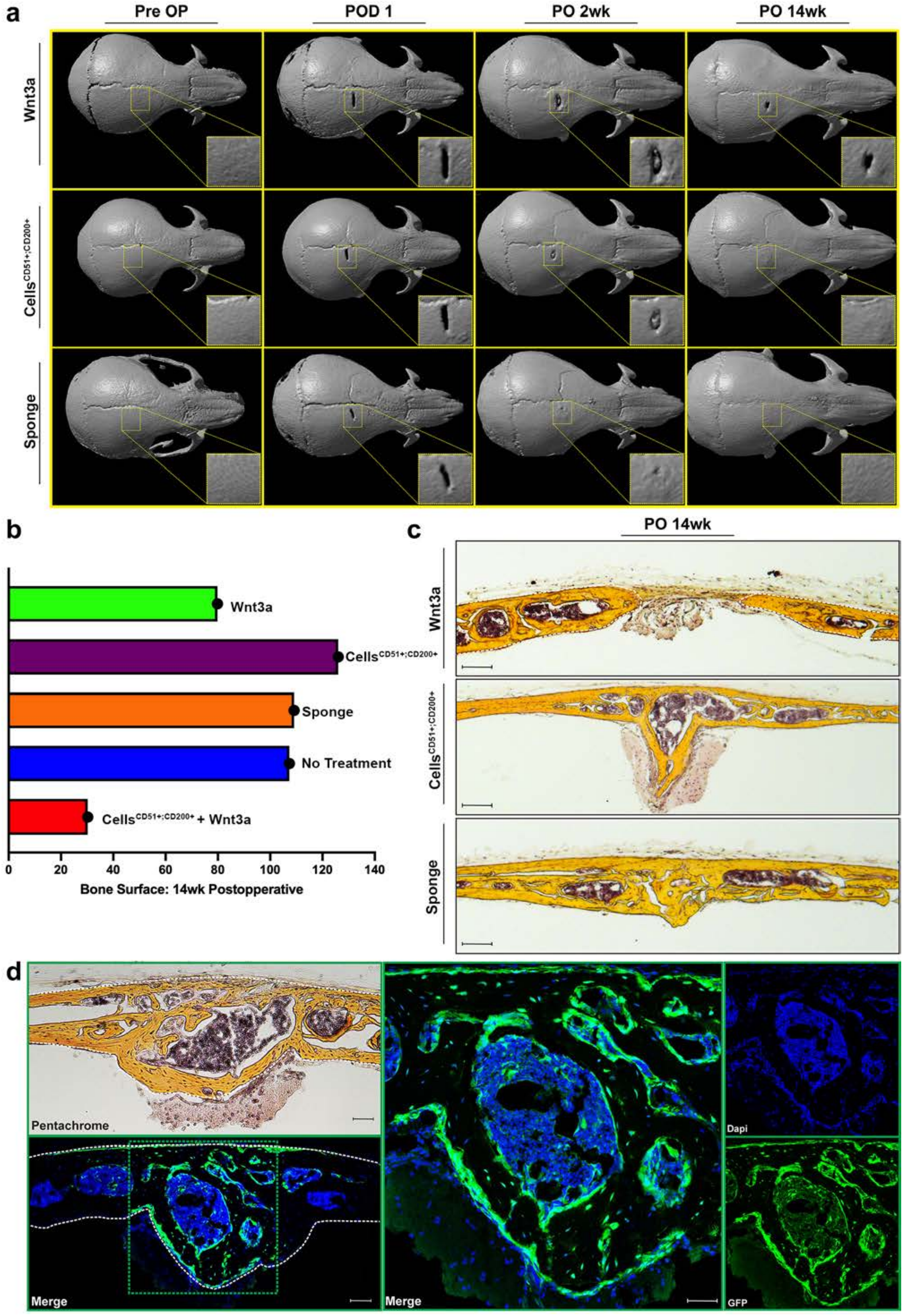

Supplementary Figure 12. Transplantation of Wnt3a Protein, $CD51^{+}$;$CD200^{+}$ Cells and Collagen Sponge alone Following Surgical Repair of Craniosynostosis. a, Time-course of micro-CT analysis of Wnt3a protein, $CD51^{+}$;$CD200^{+}$ cells and sponge alone treated COR suturectomies on pN16-18 *Twist1*$^{+/-}$ mice over a 14-week period. Wnt3a treated suturectomies do not completely refuse over the 14-week period. However, in comparison to suturectomies treated with a combination of $CD51^{+}$;$CD200^{+}$ cells and Wnt3a protein (**Figure 6b**), are trending towards closure, suggesting a delay in re-fusion but not prevention. Suturectomies treated with SSC or sponge alone, nearly refuse 2-weeks post-operatively and are completely fused by 14-weeks postoperatively. These data suggest a synergist relationship between co-transplanted $CD51^{+}$;$CD200^{+}$ cells and Wnt3a in preventing the re-fusion. n=2 animals/group, experiments were repeated 3 independent times. **b**, Quantification of COR suturectomy re-fusion by micro-CT 14-week postoperatively. Wnt3a only (green), $CD51^{+}$;$CD200^{+}$ cells only (purple), sponge only (orange), and no treatment (blue), suturectomies showed increased bone surface measurements 14-weeks postoperatively compared to suturectomies treated with a combination of $CD51^{+}$;$CD200^{+}$ cells and Wnt3a protein (red). n=1 animal/group. **c,** Movat's pentachrome staining of sagittal sections of COR suturectomies at 14-weeks showing near re-fusion of the Wnt3a only treated suturectomy and complete re-fusion of the $CD51^{+}$;$CD200^{+}$ cells and sponge alone suturectomies. Magnification 5x, scale bars: 200µm. n=2 animals/group, experiments were performed 3 independent times. **d**, wild-type GFP^{+} $CD51^{+}$;$CD200^{+}$ cells ($3x10^{5}$ cells) were loaded onto a collagen scaffold sponge and transplanted as described to monitor their presence within the ablated COR suture at 10-weeks post-operative in the absence of recombinant Wnt3a protein. Suturectomies treated with $CD51^{+}$;$CD200^{+}$ cells alone refused by 10-weeks. (top panel), Movat's pentachrome staining of sagittal sections of COR suturectomy. Magnification 10x, scale bar: 100µm. (bottom panel), confocal micrograph of a sister section of COR suturectomies, where fluorescence indicates the presence of transplanted cells within the ablated suture. Magnification 10x, scale bar: 100µm. **e,** Magnification at 20X of the boxed area in panel **d,** reveals that transplanted $CD51^{+}$;$CD200^{+}$ cells in the absence of Wnt3a participate in the re-fusion of the suturectomy by recruiting neighboring cells. Magnification 20x, scale bar: 50µm. n=2 animals/group, experiments were performed 3 independent times. Source data are provided as a Source Data file.

Supplementary Table 1

Gene	Primer Name	Primer Sequence	Amplicon Size
**Genotyping Primers**			
General CRE	Forward	GCGGTCTGGCAGTAAAAACTATC	~100 bp
	Reverse	GTGAAACAGCATTGCTGTCACTT	
	Internal Postive Control - Forward	CTAGGCCACAGAATTGAAAGATCT	~324 bp
	Internal Postive Control - Reverse	GTAGGTGGAAATTCTAGCATCATCC	
Axin-2	C-wt	AGTCCATCTTCATTCCGCCTAGC	Wild Type: ~500 bp, Mutant: ~400 bp
	C-S	AAGCTGCGTCGGATACTTGAGA	
	LacZ	TGGTAATGCTGCAGTGGCTTG	
Twist-1	Common	GGTTTCCGACTAGAGGTTTCC	Wild Type: ~221 bp, Mutant: ~500 bp
	Wild-Type Reverse	ACTGTCTGGGTCGCTGTTG	
	Mutant Reverse	CCTTCTATCGCCTTCTTGACG	

Supplementary Table 1. Genotyping Primer Sequences. Table containing genotyping primer sequences and expected outcomes used to for the following transgenic animals: General *Cre*, *Axin-2*, *Twist-1*.

Supplementary Table 2

Flow Cytometry

Antigen	Clone	Supplier	Catalog Number	Conjugate	Concentration in 100 µL ($3x10^6$ cells)
**Primary Antibodies**					
Tie2	TEK4	eBioscience	14-5987-85	Purifed	5µg
CD45	30-F11	BioLegend	103109	PE-Cy5	0.1µg
Ter119	Terr-119	Invitrogen	15-5921-81	PE-Cy5	0.1µg
Thy1.1	HIS51	eBioscience	47-0900-82	APC-eFluor 780	0.2µg
Thy1.2	53-2.1	eBioscience	47-0902-82	APC-eFluor 780	0.2µg
Ly-51	6C3	BioLegend	108311	Alexa647	0.5µg
CD105	MJ7/18	eBioscience	13-1051-82	Biotin	1 µg
CD200	OX-90	BioLegend	123802	Purifed	1 µg
CD51	RMV-7	BD Bioscience	551187	PE	1 µg
**Secondary Antibodies**					
Streptavidin	n/a	eBioscience	25-4317-82	PE-Cy7	0.2µg

Immunofluorescence

Antigen	Clone	Supplier	Catalog Number	Conjugate	Dilution
**Primary Antibodies**					
Active-β-Catenin	7E8	Millipore Sigma	05-665	Purifed	1:100
**Secondary Antibodies**					
Goat anti-Mouse	n/a	ThermoFisher	A-11001	Alexa Fluor 488	1:1000

Supplementary Table 2. Antibodies. Table containing all antibodies used for flow cytometry and immunofluorescence experiments including supplier, catalog number, conjugate and working concentration.

Supplementary Table 3

Growth Media			
DMEM GlutaMax	Gibco-Life Technologies	10566016	-
Alpha-MEM GlutaMax	Gibco-Life Technologies	32561102	-
Performance FBSA18:D30	Gibco-Life Technologies	26140-079	10%
Pen Strep	Gibco-Life Technologies	26140-079	1%
Ciprofloxacin HCL	bioWORLD	40310031-3	0.10%
**Osteogenic Differentiation Media**			
Stem Pro Osteogenesis Differnti	Gibco - Thermo Fisher Scientific	A1007201	-
Pen Strep	Gibco-Life Technologies	26140-079	1%
Ciprofloxacin HCL	bioWORLD	40310031-3	0.10%
**Chondrogenic Differentiation Media**			
Stem Pro Chondrogenesis Differ	Gibco - Thermo Fisher Scientific	A1007101	-
Pen Strep	Gibco-Life Technologies	26140-079	1%
Ciprofloxacin HCL	bioWORLD	40310031-3	0.10%
**Cell Dissociation**			
Stem Pro Accutase	Gibco	A1110501	-

Supplementary Table 3. Media. Table containing information regarding all media and reagents used for cell culture and cell differentiations experiments.

Supplementary Table 4

Reagent	Supplier	Catalog Number	Working Concentration
**Recombinant Proteins**			
Mouse Wnt3a Protein (Carrier Free)	R&D Systems	1324-WN-002/CF	150ng
Mouse sFrp-1 Protein (Carrier Free)	R&D Systems	9019-SF-025/CF	2µg
Mouse Dkk-1 Protein (Carrier Free)	R&D Systems	5897-DK-010/CF	2µg
**Small Molecules**			
SB431542	Selleckchem.com	S1067	10µM
**Cre Induction**			
Tamoxifen	Sigma-Aldrich	T5648-1G	20mg/ml
Corn Oil	Sigma-Aldrich	C8267 - 500ml	-
**Histology**			
Click-iT EdU Alexa Fluor 488 Imaging Kit	ThermoFisher	C10337	-
X-Gal	Sigma-Aldrich	10745740001	1mg/ml
Glutaraldehyde Solution (Grade II, 25% in H2O)	Sigma-Aldrich	G6257	0.20%
Formaldehyde Aqueous Solution (Paraformaldehyde Aque	ELECTRON MICROSCOPY SCIE	15710	0.4-4%
Tissue-Tek O.C.T Compound	VWR	25608-930	-
**Molecular Biology Reagents**			
TRIzol Reagent	ThermoFisher/Invitorgen	15596026	-
SuperScript III First-Strand Synthesis System	ThermoFisher/Invitorgen	18080051	-
KAPA2G Fast HotStart ReadyMix	KAPA BIOSYSTEMS	KK5609	-
HotStarTaq Plus Master Mix Kit	QIAGEN	203645	-
**RNA-Sequencing Reagents**			
miRneasy Micro Kit	Qiagen	217084	-
Ultra low input RNA kit v4	Clontech	634888	
Low Input Library Rep Kit v2	Clontech	634899	
Recombinant Rnase inhibitor (RRI)	Clontech	2313B	4 units
Triton X-100	ThermoFisher	85111	0.10%
dNTP	ThermoFisher	10297018	2.4mM
OligodT30VN (5′AAGCAGTGGTATCAACGCAGAGT/	Integrated DNA Technology	-	2.5µM
Smartscribe Reverse Transcriptase	Clontech	639538	100 units
Betaine	Sigma-Aldrich	B0300-5VL	1M
Template Switch Oligo (5′-AAGCAGTGGTATCAACGC/	Qiagen	-	1µM
1X Kapa HiFi HotStart	KAPA BIOSYSTEMS	KK2602	-
ISPCR primer (5′-AAGCAGTGGTATCAACGCAGAGT-	Integrated DNA Technology	-	0.1µM
Fragment Anakyzer High Sensitivity NGS 1-6000 Kit	Agilent	474-0500	-

Supplementary Table 4. Miscellaneous Reagents. Table containing additional information for recombinant proteins, small molecules, CRE induction, histology, molecular biology and RNA-sequencing experiments.

Supplementary Table 5

Gene	Accession Number	Species	Primer Sequence	Annealing Temp	Amplicon Size
			Genotyping Primers		
Smad6	NM_008542.3	Mouse	Fwd: TCTGCTTCGGTGGATTGCAT	57ºC	172bp
			Rev: CTTGGATTTTGCACGCACGA		
Id2	NM_010496.3	Mouse	Fwd: GATGATCGTCTTGCCCAGGT	57ºC	217bp
			Rev: TCTGGTATTCACGCTCCACC		
Bglap	L24431	Mouse	Fwd: ACCCTGGCTGCGCTCTGTCT	60ºC	443bp
			Rev: ACTATGGAAGGCTAAGGGCTCTGG		
Cdc45	AF098068.1	Mouse	Fwd: TCCAAGCCAGCATCCTTGAG	58ºC	311bp
			Rev: AGTGACACAAGAGCGTCCAG		
Huwe1	NM_021523.4	Mouse	Fwd: CCTGCCTTCAGCTCACACAT	58ºC	735bp
			Rev: ATCAGGAAGGGCAGAAATGGT		
Efnb2	NM_010111.6	Mouse	Fwd: GTTCCAGTGCTCTCCTGACTG	58ºC	955bp
			Rev: GGCCCTCCAAAGACCCATTT		
Adhc1	BC019130	Mouse	Fwd: CCACGCGTCCGTCCAAA	57ºC	457bp
			Rev: TCCTTGAAGCCTAGCGTTGG		
Dusp6	NM_026268.3	Mouse	Fwd: CGGAAATGGCGATCTGCAAG	58ºC	279bp
			Rev: AGTCGCTGCTATTCTCGTCG		
Jag1	NM_013822	Mouse	Fwd: CGCCTCAAAGAAGCGATCAG	58ºC	603bp
			Rev: GATGCGATTACGGTCGTTGC		
Ezh2	NM_001146689	Mouse	Fwd: TCCATGCAACACCCAACACA	58ºC	125bp
			Rev: AGCAGTAAGAGCAGCAGCAA		
Ptpn11	NM_001109992.1	Mouse	Fwd: CACAGAGAGAACCGTCTGGC	58ºC	710bp
			Rev: GCACGTGCTGTTCAAATGGT		
Gapdh	BC083080	Mouse	Fwd: CGGCAAATTCAACGGCACAGTCAA	60.5ºC	424bp
			Rev: CTTTCCAGAGGGGCCATCCACAG		

Supplementary Table 5. PCR Primers Sequences. Table containing PCR primer sequences used for gene expression experiments in Figure 3e-f and Supplementary Figure 3b.

Supplementary Figure 3 Raw Gel Scans:

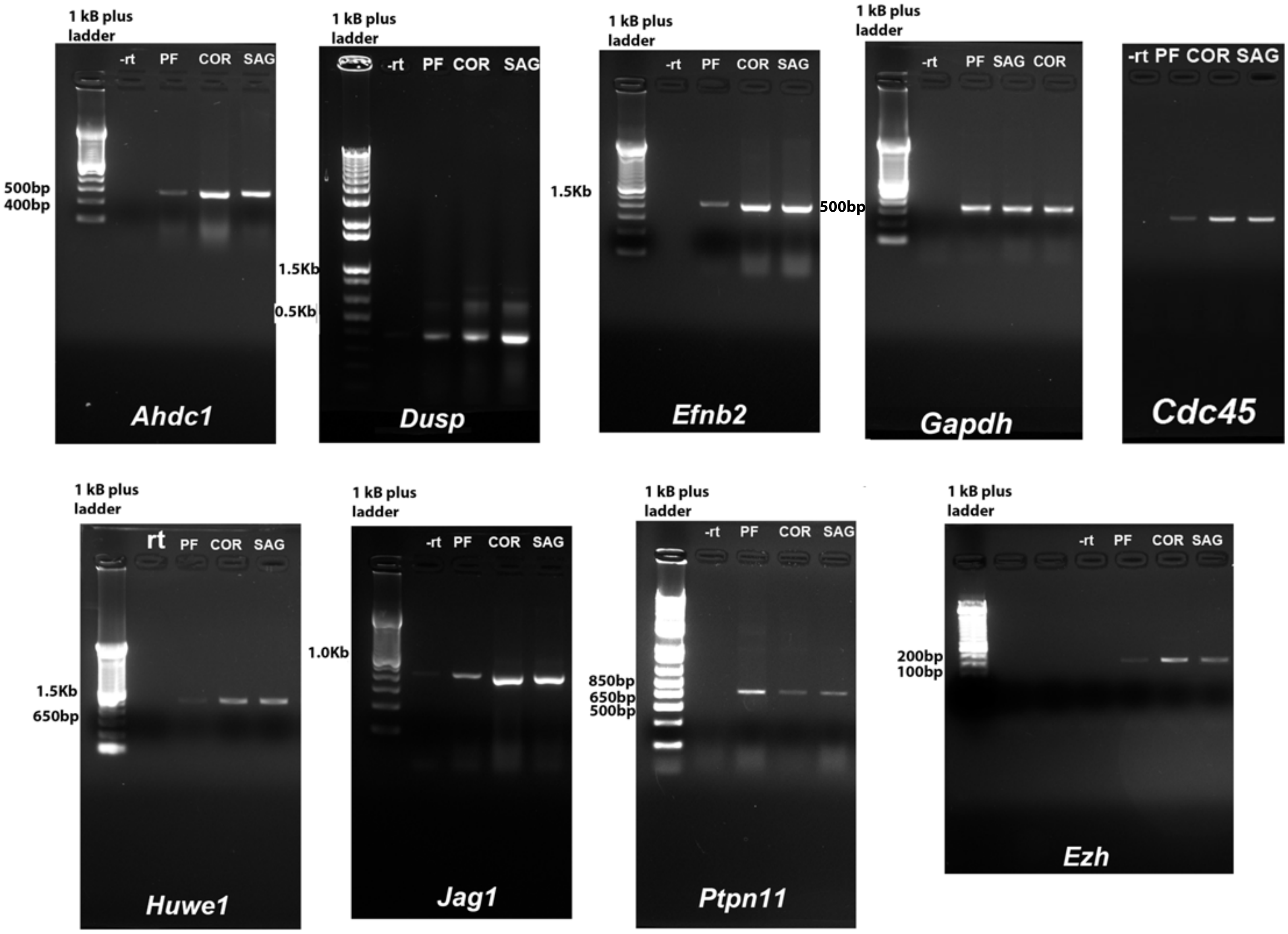

Supplement: Supplementary file 1 — Supplementary Information [file 41467_2021_24801_MOESM1_ESM.pdf]
