# Supplementary material for: Skeletal stem and progenitor cells maintain cranial suture patency and prevent craniosynostosis
Source: Nat Commun. 2021 Jul 30;12:4640. doi: 10.1038/s41467-021-24801-6 (PMC8324898; doi:10.1038/s41467-021-24801-6)
Supplement: Supplementary file 3 — Reporting Summary [file 41467_2021_24801_MOESM3_ESM.pdf]

Corresponding author(s): Natalina Quarto, Michael T. Longaker

Last updated by author(s): Jul 2, 2021

Reporting Summary

Nature Research wishes to improve the reproducibility of the work that we publish. This form provides structure for consistency and transparency in reporting. For further information on Nature Research policies, see our Editorial Policies and the Editorial Policy Checklist.

Statistics

For all statistical analyses, confirm that the following items are present in the figure legend, table legend, main text, or Methods section.

n/a	Confirmed	
☐	☒	The exact sample size (*n*) for each experimental group/condition, given as a discrete number and unit of measurement
☐	☒	A statement on whether measurements were taken from distinct samples or whether the same sample was measured repeatedly
☐	☒	The statistical test(s) used AND whether they are one- or two-sided<br>*Only common tests should be described solely by name; describe more complex techniques in the Methods section.*
☐	☒	A description of all covariates tested
☐	☒	A description of any assumptions or corrections, such as tests of normality and adjustment for multiple comparisons
☐	☒	A full description of the statistical parameters including central tendency (e.g. means) or other basic estimates (e.g. regression coefficient) AND variation (e.g. standard deviation) or associated estimates of uncertainty (e.g. confidence intervals)
☐	☒	For null hypothesis testing, the test statistic (e.g. F, t, r) with confidence intervals, effect sizes, degrees of freedom and P value noted<br>*Give P values as exact values whenever suitable.*
☒	☐	For Bayesian analysis, information on the choice of priors and Markov chain Monte Carlo settings
☒	☐	For hierarchical and complex designs, identification of the appropriate level for tests and full reporting of outcomes
☒	☐	Estimates of effect sizes (e.g. Cohen's *d*, Pearson's *r*), indicating how they were calculated

Our web collection on statistics for biologists contains articles on many of the points above.

Software and code

Policy information about availability of computer code

Data collection	No code was used for data collection
Data analysis	GraphPad Prism 8.0.0, FACS Diva (BD) v8.0.1, FlowJo 10.1r5 (TreeStar), ImageJ 2.0 (NIH), Adobe Photoshop 13.0.0, NRecon v1.7.1.6 (Bruker), CTVol v2.3.2.0 (Bruker) and CTAn v1.18.8.0+ (Bruker) software packages were used to analyze the data in this study where indicated.<br><br>Bulk RNA-seq: FastQC v0.11.5, STAR v2.5.1b, Cufflinks v2.2.1 suite, Vennt v0.8.4, Java TreeView v1.1.6r4, Cluster v3.0, scRNA-Seq: STAR v2.5.3a, Seurat13 v2.3.0, R v3.4.4, EnrichR 2.1, Ratio of Global Unshifted Entropy (ROGUE) (Liu et al., Nat Commu 2020) were used for analysis. The software packages used in this study are publicly available.

For manuscripts utilizing custom algorithms or software that are central to the research but not yet described in published literature, software must be made available to editors and reviewers. We strongly encourage code deposition in a community repository (e.g. GitHub). See the Nature Research guidelines for submitting code & software for further information.

Data

Policy information about availability of data

All manuscripts must include a data availability statement. This statement should provide the following information, where applicable:

- Accession codes, unique identifiers, or web links for publicly available datasets
- A list of figures that have associated raw data
- A description of any restrictions on data availability

All data to support the conclusions in this manuscript can be found in the figures. All source data for plots are available in the attached source data file and any other data can be requested from the corresponding authors. The mouse mm9 reference genome database was used for all RNA-seq experiments. Publicly available

data sets were obtained from Gene Expression Omnibus under the following accession numbers; Debnath et al., Nature 2018 GSE106237 and Chan et al., Cell 2015 GSE64447. All RNA-seq and scRNA-seq data generated from this study can be accessed from the Gene Expressions Omnibus (http://www.ncbi.nlm.nih.gov/geo/) using accession number GSE138882.

Field-specific reporting

Please select the one below that is the best fit for your research. If you are not sure, read the appropriate sections before making your selection.

☒ Life sciences ☐ Behavioural & social sciences ☐ Ecological, evolutionary & environmental sciences

For a reference copy of the document with all sections, see nature.com/documents/nr-reporting-summary-flat.pdf

Life sciences study design

All studies must disclose on these points even when the disclosure is negative.

Sample size	Experimental animals were used across a range of time-points and analyzed by multiple independent experiments with no fewer than 10 mice (and up to 80) per experimental time-point. Exact details of sample size, with technical and biological replicates are indicated for each experimental time-point analyzed. Sampling sizes were determined based on viability during surgical procedures and by access to the appropriately aged transgenic animals.
Data exclusions	No data were excluded from the analyses.
Replication	Analysis were reproduced in a minimum of 10 (and up to 80) animals per condition and across a minimum of two independent experiments. All attempts at replication were successful.
Randomization	Animals with appropriate genotype were randomly assigned to each experimental group.
Blinding	All data analysis and experiments with any potential for observer bias that could affect outcomes were conducted in a blinded manner, such as cell counting, micro-CT analysis, assessment of molecular treatments, clonal analysis, and group allocation during data collection. Whenever feasible, an independent investigator genotype/identified animals and/or assigned them to each group. Analysis were then carried out by a blinded investigator. Group genotype and/or treatment were revealed post analysis.

Reporting for specific materials, systems and methods

We require information from authors about some types of materials, experimental systems and methods used in many studies. Here, indicate whether each material, system or method listed is relevant to your study. If you are not sure if a list item applies to your research, read the appropriate section before selecting a response.

Materials & experimental systems

n/a	Involved in the study
☐	☒ Antibodies
☒	☐ Eukaryotic cell lines
☒	☐ Palaeontology and archaeology
☐	☒ Animals and other organisms
☒	☐ Human research participants
☒	☐ Clinical data
☒	☐ Dual use research of concern

Methods

n/a	Involved in the study
☒	☐ ChIP-seq
☐	☒ Flow cytometry
☒	☐ MRI-based neuroimaging

Antibodies

Antibodies used	The following antibodies were used for flow cytometry:<br>CD200 Purified (rat anti mouse, dilution 1:50, Biolegend, 123802, lot B288779, clone: OX-90), Qdot 605 Secondary (goat anti rat, dilution: 1:100, ThermoFisher, Q-11601MP, lot 2009239), Tie2 Purified (rat anti mouse, dilution 1:20, eBioscience, 14-5987-81, lot 2299779, clone: TEK4), CD45 PE-Cy5 (rat anti mouse, dilution 1:200, Biolegend, 103110, lot B283425. clone: 30-F11), Ter119 PE-Cy5 (rat anti mouse, dilution 1:200, eBioscience, 15-5921-81, lot 2093721, clone: TER-119), CD51 (AlphaV Integirn) PE (rat anti mouse, dilution 1:50, eBioscience, 551187, lot 8314613, clone: RMV-7), CD105 Biotin (rat anti mouse, dilution 1:50, eBioscience, 13-1051-81, lot 2189466, clone: MJ7/18), Streptavidin PE-Cy7, dilution 1:100, eBioscience, 25-4317-82, lot 2034750), Thy1.1 APC-eFuor 780, mouse anti mouse, dilution 1:100, eBioscience, 47-0900-80, lot 2142928, clone: HIS51), Thy1.2 APC-eFluor 780, rat anti mouse, dilution 1:100, eBioscience, 47-0902-82, lot 1995522, clone: 53-2.1), 6C3 APC-Alexa 647, rat anti mouse, dilution 1:100, Biolegend, 108312, lot B222435, clone: Ly-51).<br><br>The following Antibodies were used for immunofluorescence:<br>Anti-Active-β-Catenin (Anti-ABC) Antibody, clone 8E7, mouse anti mouse/rat/human, 1:100, Millipore Sigma, 05-665, lot3429115, Secondary Antibody: Alexa Fluor 488 Goat anti-Mouse IgG (H+L) , 1:1000, ThermoFisher Scientific, A-11001, lot 2140660

Validation

All primary antibodies used in this study were validated by the supplier/manufacture for use in flow cytometry or immunofluorescence applications respectively. Dilutions and concentrations were determined according to the manufacturer's recommendations in addition to the following publications referencing the isolation of the mSSCs; Identification and specification of the mouse skeletal stem cell, PMID:25594184 and Isolation and functional assessment of mouse skeletal stem cell linage, PMID 29748647.

Manufacture Validation Statements:

Tie-2 (eBioscience, 14-5987-85)
Applications Reported: This TEK4 antibody has been reported for use in flow cytometric analysis, and immunohistology staining of frozen tissue sections.
Applications Tested: The TEK4 antibody has been tested by blocking of staining with fluorochrome conjugated TEK4 on mouse bone marrow cells. This can be used at less than or equal to 1 µg per test. A test is defined as the amount (µg) of antibody that will stain a cell sample in a final volume of 100 µL. Cell number should be determined empirically but can range from 10^5 to 10^8 cells/test. It is recommended that the antibody be carefully titrated for optimal performance in the assay of interest.

Thy1.1 (eBioscience, 47-0900-82)
Applications Reported: This HIS51 antibody has been reported for use in flow cytometric analysis.
Applications Tested: This HIS51 antibody has been tested by flow cytometric analysis of rat splenocytes. This can be used at less than or equal to 0.06 µg per test. A test is defined as the amount (µg) of antibody that will stain a cell sample in a final volume of 100 µL. Cell number should be determined empirically but can range from 10^5 to 10^8 cells/test. It is recommended that the antibody be carefully titrated for optimal performance in the assay of interest.

Thy1.2 (eBioscience, 47-0900-82)
Applications Reported: This 53-2.1 antibody has been reported for use in flow cytometric analysis.
Applications Tested: This 53-2.1 antibody has been tested by flow cytometric analysis of mouse splenocytes. This can be used at less than or equal to 0.125 µg per test. A test is defined as the amount (µg) of antibody that will stain a cell sample in a final volume of 100 µL. Cell number should be determined empirically but can range from 10^5 to 10^8 cells/test. It is recommended that the antibody be carefully titrated for optimal performance in the assay of interest.

CD105 (eBioscience, 13-1051-82)
Applications Reported: The MJ7/18 antibody has been reported for use in flow cytometric analysis.
Applications Tested: The MJ7/18 antibody has been tested by flow cytometric analysis of bEnd.3 cells and mouse splenocytes. This can be used at less than or equal to 0.5 µg per test. A test is defined as the amount (µg) of antibody that will stain a cell sample in a final volume of 100 µL. Cell number should be determined empirically but can range from 10^5 to 10^8 cells/test. It is recommended that the antibody be carefully titrated for optimal performance in the assay of interest.

Terr-119 (Invitrogen/eBioscience, 15-5921-81)
Applications Reported: The TER-119 antibody has been reported for use in flow cytometric analysis.
Applications Tested: The TER-119 antibody has been tested by flow cytometric analysis of mouse splenocytes and bone marrow cells. This can be used at less than or equal to 0.25 µg per test. A test is defined as the amount (µg) of antibody that will stain a cell sample in a final volume of 100 µL. Cell number should be determined empirically but can range from 10^5 to 10^8 cells/test. It is recommended that the antibody be carefully titrated for optimal performance in the assay of interest.

CD51 (BD Bioscience, 551187)
Reactivity: Mouse (QC Testing)
Application:Flow cytometry (Routinely Tested)

CD200 (BioLegend, 123802) CD45 (BioLegend, 103109) and 6C3 (BioLegend, 108311)
Each lot of this antibody is quality control tested by immunofluorescent staining with flow cytometric analysis. For flow cytometric staining, the suggested use of this reagent is ≤ 0.25 µg per 106 cells in 100 µl volume. It is recommended that the reagent be titrated for optimal performance for each application. (https://www.biolegend.com/protocols/cell-surface-flow-cytometry-staining-protocol/4283/)

Active-β-Catenin (Millipore Sigma, 05-665)
Application: Anti-Active-β-Catenin (anti-ABC) Antibody, clone 8E7 is a well characterized Mouse Monoclonal Antibody. This highly published mAb also known as Anti-Catenin beta-1 readily detects beta-Catenin & has been validated in FC, ICC, IHC, IHC(P) & WB.
Application Notes: Immunocytochemistry: This antibody was used in immunocytochemistry and showed positive staining on the membrane and cytosol.This antibody has also been reported by an independent laboratory to show positive immunostaining for beta-catenin in LiCl-treated 293T cells fixed with methanol (Staal, Frank J. T., 2002). Flow Cytometry: This antibody was used in flow cytometry at an optimal 1 µg/mL concentration. Immunohistochemistry: This antibody was used in immunohistochemistry on a colorectal carcinoma tissue array at a 1:300 dilution. This antibody has also been reported by an independent laboratory to detect beta-catenin in mouse embryo sections (Van Noort, M., 2002).

Quality Assurance: Routinely evaluated by immunoblot on RIPA lysates from A431 cells. Western Blot Analysis: 0.2-2 µg/mL of this antibody detected β-catenin in RIPA lysates from A431 cells.

Animals and other organisms

Policy information about studies involving animals; ARRIVE guidelines recommended for reporting animal research

Laboratory animals	Experiments using animals were performed on the following mice strains. C57BL/6, CD-1 IGS, Twist1+/-, Axin2LacZ/+, Axin2LacZ/+:Twist1+/-, B6 ACTb-EGFP, ActinCreERT2:R26Rainbow, Twist1+/-:ActinCreERT2:R26Rainbow, Axin2LacZ/+:ActinCreERT2:R26Rainbow. Age of animals vary by experiment, ranging from post-natal day 3 to 6 months of age. Both males and female animals used in this study. Details of breeding strategy and genotyping are included in the Materials and Methods sections under “Animals”.
Wild animals	No wild animals were used in this study.
Field-collected samples	No field-collected samples were used in this study.
Ethics oversight	All experiments using animals were performed in accordance with Stanford University Animal Care and Use Committee guidelines. The Administrative Panel on Laboratory Animal Care (APLAC) approved all protocols and procedures regarding animal care, genotyping and all surgical procedures. (Stanford APLAC protocol 8397 and 21067)

Note that full information on the approval of the study protocol must also be provided in the manuscript.

Flow Cytometry

Plots

Confirm that:

- [x] The axis labels state the marker and fluorochrome used (e.g. CD4-FITC).
- [x] The axis scales are clearly visible. Include numbers along axes only for bottom left plot of group (a 'group' is an analysis of identical markers).
- [x] All plots are contour plots with outliers or pseudocolor plots.
- [x] A numerical value for number of cells or percentage (with statistics) is provided.

Methodology

Sample preparation	Posterior Frontal, Sagittal, Coronal sutures and Tibial Growth Plate were carefully dissected on ice with the aid of a dissection microscope. Tissue was gently minced and serially digested in a collagenase digestion buffer supplemented with DNase incubated in a 37° water bath for 10 min followed by 2, 25min digestions in an orbital shaker at 275rpm at 37°. Dissociated cells were filtered though a 70µm filter and digestions pooled together and pelleted at 1350rpm at 4°, resuspended in FACS buffer, and stained with flourochrome-conjugated antibodies against CD200, Tie2, CD45, Ter119, CD51, CD105, Thy1.1, Thy1.2, and 6C3 for 20min under light agitation. Phosphatidylinositol (PI) staining was performed to exclude dead cells. Details of cell dissociation and flow cytometry are included in the Materials and Methods section under “In-vivo suture harvest and cells dissociation, Flow Cytometry” and Supplementary Figure 1.
Instrument	Flow cytometry was performed on the FACS Aria II in the Lorey Lokey Stem Cell Institute Shared FACS Facility.
Software	Data was analyzed using the BD FACSDiva software or imported from the FACS Aria II into the FlowJo software for analysis. Analysis was completed using the gating strategy described in the material and methods, "Flow Cytometry" and Supplementary Figure 1.
Cell population abundance	Prospective cell populations were double sorted on purity and assessed for they’re functional ability in in-vitro assays and purity assessed by semi-quantitative PCR analysis.
Gating strategy	Single cell were first gated using FSC and SSC parameters. Hematopoietic (CD45-) and dead cells (PI+) were gated out and the remaining population was fractionated based on the following immunophenotype: CD45-, Ter119-, Tie2-, Thy1.1-, Thy1.2-, 6C3-, CD105-, CD51+, CD200+.) Negative “unstained with PI” and fluorescence minus one (FMO) control samples were used to excluded dead cells and discriminate between positive and negative populations.

- [x] Tick this box to confirm that a figure exemplifying the gating strategy is provided in the Supplementary Information.
