# Supplementary material for: Skeletal stem and progenitor cells maintain cranial suture patency and prevent craniosynostosis
Source: Nat Commun. 2021 Jul 30;12:4640. doi: 10.1038/s41467-021-24801-6 (PMC8324898; doi:10.1038/s41467-021-24801-6)
Supplement: Supplementary file 4 — Source data [file 41467_2021_24801_MOESM4_ESM.pdf]

**Figure 1b - Time Course**

| Post-Natal Day | 3 | 5 | 7 | 9 | 11 | 15 | 17 |
|---|---|---|---|---|---|---|---|
| PF | 6.555 | 5.87 | 6.161 | 1.776 | 1.113 | 0.431 | 0.26 |
| | 9.541 | 8.687 | 2.636 | 1.598 | 0.819 | 0.171 | 0.25 |
| | 9.519 | | | | | | 0.141 |
| | 9.656 | | | | | | 0.255 |
| | 11.983 | | | | | | |
| | 6.179 | | | | | | |
| | | | | | | | |
| SAG | 5.632 | 3.261 | 3.391 | 2.012 | 3.242 | 4.939 | 2.82 |
| | 7.138 | 5.49 | 4.975 | 4.641 | 2.431 | 8.207 | 3.06 |
| | 5.858 | | | | | | 12.04 |
| | 9.495 | | | | | | 6.44 |
| | 10.241 | | | | | | |
| | 5.665 | | | | | | |
| | | | | | | | |
| COR | 2.909 | 1.939 | 3.826 | 2.84 | 6.16 | 2.4 | 4.433 |
| | 3.643 | 4.325 | 4.167 | 5.85 | 1.22 | 1.151 | 1.56 |
| | 3.116 | | | | | 7.596 | 10.42 |
| | 3.657 | | | | | | 2.466 |
| | 3.542 | | | | | | |
| | 3.665 | | | | | | |
| | | | | | | | |
| GP | 6.872 | 4.522 | 4.203 | 4.548 | 3.323 | 2.433 | 4.111 |
| | 6.051 | 6.17 | 7.182 | 9.392 | 9.645 | 5.884 | 7.84 |
| | 9.543 | | | | | | 2.5 |
| | 5.345 | | | | | | |
| | 9.521 | | | | | | |
| | 6.419 | | | | | | |

| | |
|---|---|
| Unpaired t test | pN17: PF vs SAG |
| P value | 0.0343 |
| P value summary | * |
| Significantly differ | Yes |
| | |
| Unpaired t test | pN17: PF vs COR |
| P value | 0.0147 |
| P value summary | * |
| Significantly differ | Yes |
| | |
| Unpaired t test | pN17: PF vs GP |
| P value | 0.0179 |
| P value summary | * |
| Significantly differ | Yes |

**Figure 3b - Twist vs Wt**

| Wild-Type | $Twist1^{+/-}$ |
|---|---|
| 978 | 554 |
| 948 | 537 |
| 1534 | 219 |
| 3112 | 118 |
| | |
| | |
| Unpaired t test | |
| P value | 0.0482 |
| P value summary | * |
| Significantly different (P < | Yes |

**Figure 3d - iSmad6**

| SAG + SB431542 | SAG Control |
|---|---|
| 21 | 45 |
| 19.4 | 48 |
| 22.5 | 44.5 |
| | |
| Unpaired t test | |
| P value | <0.0001 |
| P value summary | **** |
| Significantly different (P < | Yes |

**Figure 3e - Id2**

| SAG + SB431542 | SAG Control |
|---|---|
| 53 | 15 |
| 47.5 | 17.1 |
| 48 | 19.5 |
| | |
| Unpaired t test | |
| P value | 0.0001 |
| P value summary | *** |
| Significantly different (P < | Yes |

**Figure 3f - Bglap**

| SAG + SB431542 | SAG Control |
|---|---|
| 19.5 | 4.2 |
| 17.9 | 3.88 |
| 19.6 | 5.6 |
| | |
| Unpaired t test | |
| P value | <0.0001 |
| P value summary | **** |
| Significantly different (P < | Yes |

**Figure 3g - SB Treatment Vs Control**

| SAG + SB431542 | SAG Control |
|---|---|
| 612 | 3876 |
| 839 | 2928 |
| 388 | 1181 |
| | 2711 |
| | |
| Unpaired t test | |
| P value | 0.0478 |
| P value summary | * |
| Significantly differe | Yes |

**Figure 4c - Wnt3a Treatment**

| No Treatment | Wnt3a | PBS |
|---|---|---|
| 0.62 | 2.96 | 0.67 |
| 0.6 | 2.82 | 0.72 |
| 0.41 | 1.41 | 1.23 |
| 1.22 | 3.78 | |
| 2.26 | 3.4 | 1.4 |
| 2 | 5.14 | 4.41 |
| 0.21 | | 0.17 |
| | | |
| Unpaired t test - NT v Wnt3a | | |
| P value | 0.0025 | |
| P value summary | ** | |
| Significantly differen | Yes | |
| | | |
| Unpaired t test - NT v PBS | | |
| P value | 0.569 | |
| P value summary | ns | |
| Significantly differen | No | |
| | | |
| Unpaired t test - Wnt3a Vs PBS | | |
| P value | 0.046 | |
| P value summary | * | |
| Significantly differen | Yes | |

**Figure 4f - Dkk-1/sFRP-1 Treatment**

| No Treatment | Dkk-1/sFrp-1 | PBS |
|---|---|---|
| 7.3 | 4 | 5.56 |
| 7.81 | 4.3 | 7.07 |
| | | |
| Unpaired t test - Dkk/sFRP vs NT | | |
| P value | 0.0075 | |
| P value summary | ** | |
| Significantly different ( | Yes | |
| | | |
| Unpaired t test - PBS vs NT | | |
| P value | 0.26 | |
| P value summary | ns | |
| Significantly different ( | No | |
| | | |
| Unpaired t test - PBS vs Dkk/sFRP | | |
| P value | 0.1066 | |
| P value summary | ns | |
| Significantly different ( | No | |

**Figure 5b - Twist:Axin Rescue SSCs**

| Wild-Type | *Axin2*$^{LacZ/+}$ | *Twist1*$^{+/-}$:*Axin*$^{LacZ/+}$ | *Twist1*$^{+/-}$ |
|---|---|---|---|
| 2.4 | 1.87 | 2.18 | 1.37 |
| 1.151 | 2.69 | 3.38 | 1.34 |
| 7.59665003 | 3.033 | 3.597 | 1.24 |

| | |
|---|---|
| Unpaired t test - WT v Twist | |
| P value | 0.291 |
| P value summary | ns |
| Significantly diffe | No |
| | |
| Unpaired t test - WT vs Axin | |
| P value | 0.5861 |
| P value summary | ns |
| Significantly diffe | No |
| | |
| Unpaired t test - WT vs Twist:Axin | |
| P value | 0.7593 |
| P value summary | ns |
| Significantly diffe | No |
| | |
| Unpaired t test - Axin vs Twist | |
| P value | 0.025 |
| P value summary | * |
| Significantly diffe | Yes |
| | |
| Unpaired t test - Axin vs Twist | |
| P value | |
| P value summary | |
| Significantly different (P < 0.05)? | |
| | |
| | |
| Unpaired t test - Twist:Axin vs Twist | |
| P value | 0.0172 |
| P value summary | * |
| Significantly diffe | Yes |

**Figure 6c - Bone Surface Quantification**

| Post Operative | No Treatment | Wnt3a + SSCs |
|---|---|---|
| POD 1 | 56.08 | 54.248 |
| PO 2wk | 76.72 | 44.216 |
| PO 4wk | 96.45 | 34.546 |
| PO 6wk | 98.67 | 22.653 |
| PO 8wk | 98.39 | 22.295 |
| PO 10wk | 99.87 | 22.288 |
| PO 14wk | 107.34 | 30.243 |

**Supplementary Figure 2c - CFU Assay**

| PF | SAG | COR |
|---|---|---|
| 8 | 34 | 19 |
| 13 | 36 | 19 |
| 10.5 | 35 | 18 |
| | | |
| Unpaired t test PF vs SAG | | |
| P value | <0.0001 | |
| P value su | **** | |
| Significantl | Yes | |
| | | |
| Unpaired t test SAG vs COR | | |
| P value | <0.0001 | |
| P value su | **** | |
| Significantl | Yes | |
| | | |
| Unpaired t test PF vs COR | | |
| P value | 0.0053 | |
| P value su | ** | |
| Significantl | Yes | |
| | | |
| Unpaired t test PF vs GP | | |
| P value | 0.0365 | |
| P value su | * | |
| Significantl | Yes | |
| | | |
| Unpaired t test Sag vs GP | | |
| P value | 0.0004 | |
| P value su | *** | |
| Significantl | Yes | |
| | | |
| Unpaired t test Cor vs GP | | |
| P value | 0.4639 | |
| P value su | ns | |
| Significantl | No | |

**Supplementary Figure 7a, ii - Xgal Quantification**

| Wnt3a | No Treament |
|---|---|
| 14 | 7 |
| 13 | 4 |
| 8 | 0 |
| 7 | |
| 11 | |

| | |
|---|---|
| Unpaired t test | |
| P value | 0.0254 |
| P value sum | * |
| Significantly | Yes |

**Supplementary Figure 7b, ii - Xgal Quantification**

| Dkk-1/sFrp-1 | No Treatment |
|---|---|
| 4 | 10 |
| 2 | 11 |
| 2 | 12 |
| 3 | 8 |
| 2 | 6 |

| | |
|---|---|
| Unpaired t test | |
| P value | 0.0004 |
| P value sum | *** |
| Significantly | Yes |

**Supplementary Figure 7c - Wild-type Vs Axin2(homo) PF sutures**

| Wild-Type | *Axin2*$^{LacZ/LacZ}$ |
|---|---|
| 0.14 | 1.73 |
| 0.39 | 0.974 |
| 0.4314935 | 1.44 |
| 0.171308 | |

| | |
|---|---|
| Unpaired t test | |
| P value | 0.003 |
| P value sum | ** |
| Significantly | Yes |

**Supplementary Figure 7d, ii - Quantification of Clones**

| No Treatmen | Wnt3a | PBS |
|---|---|---|
| 4 | 11 | 4 |
| 5 | 11 | 4 |
| 5 | 9 | 3 |

| | |
|---|---|
| Unpaired t test - NT vs Wnt3a | |
| P value | 0.0016 |
| P value sum | ** |
| Significantly | Yes |
| | |
| Unpaired t test - NT vs PBS | |
| P value | 0.1012 |
| P value sum | ns |
| Significantly | No |
| | |
| Unpaired t test - Wnt3a Vs PBS | |
| P value | 0.0009 |
| P value sum | *** |
| Significantly | Yes |

**Supplementary Figure 7e, ii - Quantification of Proliferating Cells**

| No Treatment | Wnt3a |
|---|---|
| 8.945 | 22.695 |
| 12.035 | 26.85 |
| 14.05 | 32.13 |

| | |
|---|---|
| Unpaired t test - No Treatment vs Wnt3a | |
| P value | 0.0075 |
| P value summary | ** |
| Significantly differe | Yes |

**Supplementary Figure 7f, ii - Quantification of Clones**

| No Treatment | Dkk-1/sFrp-1 | PBS |
|---|---|---|
| 9 | 2 | 9 |
| 12 | 2 | 12 |
| 9 | 1 | 9 |

| | |
|---|---|
| Unpaired t test - NT vs Dkk/sFRP | |
| P value | 0.0014 |
| P value summary | ** |
| Significantly differe | Yes |
| | |
| Unpaired t test - Dkk/sFrp vs PBS | |
| P value | 0.0014 |
| P value summary | ** |
| Significantly differe | Yes |
| | |
| Unpaired t test - NT vs PBS | |
| P value | >0.9999 |
| P value summary | ns |
| Significantly differe | No |

**Supplementary Figure 8d - Rainbow Quantification**

| *Twist1*$^{+/-}$*:ActinCre*$^{ERT2}$*:Rainbow*$^{+/+}$ | *Axin2*$^{LacZ/+}$*:ActinCre*$^{ERT2}$*:Rainbow*$^{+/+}$ | *ActinCre*$^{ERT2}$*:Rainbow*$^{+/+}$ |
|---|---|---|
| 4 | 10 | 4 |
| 5 | 8 | 6 |

| | | |
|---|---|---|
| Unpaired t test - Twist:rainbow vs Axin:rainbow | | |
| P value | 0.0565 | |
| P value summary | ns | |
| Significantly different (P < 0.05)? | No | |
| | | |
| Unpaired t test Twist:rainbow vs Actin:Rainbow | | |
| P value | 0.6985 | |
| P value summary | ns | |
| Significantly different (P < 0.05)? | No | |
| | | |
| Unpaired t test Axin:rainbow vs Actin:Rainbow | | |
| P value | 0.1056 | |
| P value summary | ns | |
| Significantly different (P < 0.05)? | No | |

| Supplementary Figure 12b - Quantification of COR suturectomy re-fusion by micro-CT | | | | | |
|---|---|---|---|---|---|
| Post OP Week | SSCs + Wnt3a | NT | Wnt3a | SSCs | Sponge |
| 14 | 30.243 | 107.34 | 79.84 | 126.04 | 109.16 |
